# What matters for the scalability of prejudice reduction programs and interventions? A Delphi study

**DOI:** 10.1186/s40359-022-00814-8

**Published:** 2022-04-25

**Authors:** Wing Hsieh, Rebecca Wickes, Nicholas Faulkner

**Affiliations:** 1 BehaviourWorks Australia, Monash Sustainable Development Institute, Monash University, Melbourne, Australia; 2 School of Social Sciences, Monash University, Melbourne, Australia

**Keywords:** Prejudice, Scaling, Scalability, Social science programs, Intervention design

## Abstract

**Background:**

In many countries, policy makers and practitioners turn to prejudice reduction programs and interventions to tackle prejudice in the community. However, successfully addressing prejudice requires an effective intervention that can scale to match the broad span of the problem. The scalability assessment frameworks from health sciences have varying emphasis on four categories—intervention, delivery, costs, and context. For example, the high-level factors in the two Milat et al. scalability assessments are weighted towards details of the intervention (Milae et al. in Health Promot Int 28(3):285–981, 2013; Health Res Policy Syst 2:1–17, 2020). Investigation into scalability, specific to prejudice reduction, is necessary to understand how scalability frameworks apply in a different discipline.

**Methods:**

Using a Delphi approach—a structured method to obtain consensus from experts (Milae et al. Health Promot Int 28(3):285–981, 2013; Linstone and Turoff in The Delphi method—techniques and applications, Addison-Wesley, 1975; de Meyrick in Health Educ 103(1):7–16, 2003)—to bring together 16 prejudice reduction experts from multiple sectors including NGOs, private, government and academia, we developed a scalability assessment framework of criteria that are important for the successful scaling of prejudice interventions. We then applied that framework to exemplars of prejudice reduction interventions published in the academic literature.

**Results:**

For prejudice reduction interventions, contextual factors are key considerations for successful scaling. Commonly used prejudice reduction intervention approaches like contact, whether face-to-face or online, can have limited scalability.

**Conclusions:**

To reduce prejudice there needs to be consideration of scalability. This paper presents a first-of-its-kind framework for assessing scalability for prejudice reduction interventions. Applying the empirically developed framework to actual interventions demonstrated that for interventions to be effective and scalable, a greater focus on approaches beyond face-to-face contact is required.

## Background

Over the past six decades, prejudice reduction interventions have attempted to reduce prejudice and discrimination across a range of settings [1]. The extent to which these initiatives reduce prejudice in real-world field settings is mixed [1–3]. Furthermore, little is known about whether these interventions can be scaled to reach broad populations.

For prejudice reduction programs to have widespread impact, they must be scalable and effective in changing prejudicial attitudes and discriminatory behaviour. The scaling of interventions brings its own set of challenges. What works in a specific setting may not work in a wider community intervention [4]. Without understanding what specifically makes a prejudice reduction intervention scalable, efforts to tackle prejudice are not optimised and the reach of interventions remains limited.

The challenges faced in social psychology parallel the challenges of bringing health sciences interventions into practice. As early as the 1940s, it was observed that health sciences interventions conducted in real-world clinical settings did not produce the same successful outcomes as that observed in controlled settings [5–8]. This sparked the birth of implementation science—“the scientific study of methods to promote the systematic uptake of research findings and other evidence-based practice into routine practice and, hence, to improve the quality and effectiveness of health services” [9]. Studies began appearing in the 1980s highlighting that implementation processes in the health sciences can influence real-world impact [6, 7]. The science emphasised that having evidence of what works is not enough to have a real-world impact because it is also necessary to pay attention to the dissemination and implementation of effective interventions to increase reach, adoption, and use [5, 8]. From that point, implementation science has moved towards the greater systematisation of frameworks and approaches intending to ensure that effective interventions can have a real-world impact [6, 7, 10, 11].

In the health discipline, several scalability assessments have detailed appropriate strategies for scaling health-related interventions that also consider the broader suite of “real world” factors. The health sciences have critically engaged with the concept of scalability in health interventions—both in terms of general principles as well as applied to specific programs [12–17]. In general, the scalability assessment frameworks from health sciences contain factors that can be grouped into four categories—intervention, delivery, costs, and context. Across the four categories, the emphasis of the high-level factors varied across the assessments. For example, the high-level factors in the two Milat et al. scalability assessments are weighted towards details of the intervention. In contrast, in Zamboni et al. [13] the high-level factors are concentrated in the context, covering organisational, community, and socio-political contexts. Table 1 provides a summary of the factors relevant to scalability that are included in these models from health science.

**Table 1:** Comparison of health scalability assessment frameworks

High-level categories	Milat et al. [12]	Milat et al. [18]	Zamboni et al. [13]
Intervention	Effectiveness, adoption and reach Evaluation approach	Evidence of effectiveness Reach and acceptability The intervention	Attributes of the innovation/intervention
Delivery	Intervention delivery Workforce, technical and organisational resources required	Fidelity and adoption Delivery setting and workforce Implementation infrastructure	Scale-up strategy Attributes of the implementers
Costs	Cost considerations	Intervention costs and benefits	–
Context	Contextual factors	Strategic and political context	Supportive organisational culture and leadership Attributes of adopting community Socio-political context
Other	–	The problem Sustainability	–

The factors that lead to the successful scaling of health interventions are potentially linked to those that may be relevant for scaling social science interventions. Yet, no study has considered whether there are important differences that may facilitate or hinder scaling prejudice reduction interventions. A recent meta-analysis of prejudice reduction interventions tested in field experiments revealed that not a single study materially considered scalability [19]. There are plausible reasons for differences in scaling between the two disciplines. The settings, actors, and broader political contexts that health interventions operate may manifest differently in prejudice reduction interventions. For example, while many health interventions typically experience bi-partisan support (e.g., an information campaign on sun safety), prejudice reduction initiatives can be politically polarising (e.g., proposal for religious discrimination legislation).

Scalability research is in its infancy in social sciences and only recently have scholars begun to develop the theoretical tools necessary to scale up interventions in field settings. The recent work of Al-Ubaydli et al. [20] is a cogent example of a holistic approach to scaling social science research in the real world. The model Al-Ubaydli and colleagues proposed pointed to three main barriers to scalability in small-scale, successful interventions: inference, representativeness of the population, and representativeness of the situation [20, 21]. First, the authors suggest that there may be issues with the reliability of the evidence, due to the lack of replication of the effects, insufficient power in the studies, and incorrect interpretation of the p-value of results. Second, the experimental population may not be generalisable or representative of the broader population. This could arise if recruitment is conducted to either reduce costs or maximise effect size, rather than represent the wider population. Other reasons for lack of representativeness included selection bias, non-random compliance, and attrition, or diseconomies of scale such that costs are higher at scale. Finally, Al-Ubaydli et al. [20] argue that intervention success may be entirely dependent on the particular context. For example, it could be harder to manage program fidelity and dosage at scale where the context is substantially different. Additionally, for those programs successfully scaled, political opposition may limit the program’s effectiveness.

The model proposed by Al-Ubaydli et al. [20] provides a starting point for thinking about scalability in the social sciences. However, there may be additional practical considerations that are not captured in Al-Ubaydli et al.’s model, as the model is mostly focussed on considerations that impact evidence quality and generalisability. According to Supplee and Kane [22], whether a program scales also depends on a range of additional factors such as leadership, maintenance of relationships, policy windows, financial resources, and political promises. They opined that scale-up cannot be solely contingent on the quality of evidence.

The messiness of real-world settings and the possible barriers that (non-rational) behaviour creates for the scalability of interventions have been noted by other scholars. Drawing from behavioural psychology, McConnell [23] proposed that deliberate effort was required to create universal acceptance by policymakers for using an evidence-based approach to scaling. This is a separate issue from the quality of evidence. Consideration is also needed to address the “social validity” of programs and interventions, the social appropriateness of procedures, and the social importance of effects. McConnell [23] raised an illustrative example of taking into deliberation “customer preferences”, a practice that he believes is more common in business than in academia as a way to build social validity of an intervention. These subjective contextual factors are beyond the scope of an economic model but are considered to be very relevant to the success of scaling up and should be given consideration if real world scalability is the objective. Thus, this model also is of limited applicability for scaling prejudice reduction interventions in the real world.

To advance social psychology’s efforts in reducing prejudice, an implementation science revolution is vital. The evidence of what works needs to be implemented in the real world at scale. As a discipline, social psychology needs to think deliberately about how research in controlled laboratory settings or at a small scale can translate into broader real-world impact.

Academic research on prejudice reduction interventions has centred on their effectiveness, not their scalability. This has led to a concentrated focus on initiatives that are grounded in contact theory. However, the scalability of this approach is questionable. Contact is a relatively “high-touch” intervention and recruitment can be difficult as people may be unwilling to meet outgroup members [24, 25]. To successfully tackle prejudice across communities, a framework for understanding the principles of scalability specifically for prejudice reduction interventions is necessary.

This article provides an examination of the considerations for the successful scaling of prejudice reduction interventions and programs. Using a Delphi approach—a structured method to obtain consensus from experts, representing academics, not-for-profit, private, and public sectors—this paper provides an original framework for the successful scaling of prejudice reduction interventions. This framework comprehensively covers the relevant considerations for scaling to help those working in prejudice reduction to think through whether an intervention is likely to scale. Further, it also applies as factors that need to be addressed to give an intervention the best opportunity for scaling. To this end, the specific aims of this study were: (1) to define scalability as understood in the context of prejudice reduction interventions, (2) to develop a framework for scalability designed for prejudice reduction interventions, and (3) to demonstrate the application of the framework to several exemplar prejudice reduction interventions tested in the field under “gold standard” randomised control trial conditions. In developing and applying a scalability assessment to popular prejudice reduction approaches, we uncover an understanding of the reach and impact of those interventions and consequences for the future direction of research in prejudice reduction.

## Method

This study used a Delphi approach, whereby multiple rounds of data collection were used to gain consensus across a group of experts [12, 26, 27]. The Delphi method uses structured group communication in multiple rounds that enables a group of individuals as a whole to reach a consensus on complex problems [26, 28]. A review of studies using the Delphi approach uncovered hundreds of studies across economics, health and psychology databases [29]. It is a particularly useful design when dealing with multiple expert groups who may be difficult to bring together in one plenary session. As the method is used to draw insights from preeminent experts on a particular subject, the typical number of participants is 12–14 [12, 30–32]. This approach was suitable for examining the scalability of prejudice reduction where there were many actors such as practitioners, government policymakers, human resource teams in large organisations, and academics, across a few different sectors who would be difficult to bring together in a plenary session given their geography and seniority but all contribute deep expertise on the subject matter from their individual experiences and perspectives.

### Data collection and analysis

In line with best practice for the Delphi methodological approach [12], two rounds of data collection were used. The first round involved one-on-one semi-structured interviews with experts to explore issues relevant to the topic of scalability. Our prejudice reduction experts (PREs) were asked questions about how they would define scalability, and their experience with scaling programs and interventions. Transcripts of interviews were coded by the lead author into highlevel themes and a definition of scalability was formed using a thematic analysis approach [33]. These high-level themes were reviewed by the PREs in the second-round survey. The feedback from the survey was used to refine the list of factors associated with successful scaling. Following this refinement and further reflection by the authors on how the themes would apply in practice, the themes were organised into the framework presented. Interviews were conducted from July to September 2019. This was followed by the survey, sent via email, in October 2019.

#### Recruitment

PREs were selected based on their experience in prejudice reduction and related programs, interventions, or research, broadly defined to capture a range of relevant expertise. To be shortlisted, PREs needed to have direct experience in the design, implementation, evaluation, and scaling of prejudice reduction programs and interventions. They needed to have worked in the relevant area over many years, which meant that they tended to be at least at senior management levels. Purposive sampling was used to ensure that the PREs represented a variety of key sectors including government, community organisations, private sector, and academia.

A shortlist of relevant experts was identified by the research team by referring to conference attendance lists, personal knowledge, and networks. Experts were invited to participate in the study by the lead author via email or LinkedIn messages. Additional participants were added through snowball sampling. In line with previous Delphi studies, we aimed to recruit around 12–14 experts [12, 30, 31]. Other qualitative studies which used interview-based techniques have found that the point of saturation is typically at 12 participants and that most themes will be revealed at six participants [34].

### Application of framework: proof of concept

To demonstrate the applicability of the framework to actual interventions, the lead author selected three prejudice reduction interventions. These exemplar interventions were selected on the basis that they have relatively large effect size compared to other interventions in the same approach category, according to a recent meta-analysis of prejudice reduction field experiments [19]. Two of these interventions were based on the frequently researched approach of contact—one is based on in-person contact and the other was a form of electronic contact [35, 36]. The third intervention was based on a relatively novel approach, perceived variability [37]. These interventions are considered to be common forms within their theoretical approach category. We applied a code system that indicated for each criterion in our scalability framework whether the intervention was: (*) unlikely to meet the criterion; (**) likely to meet the criterion but may need to consider specifics; or (***) very likely to meet the criterion. As this was a hypothetical exercise devoid of a specific context for consideration, the context factors were not able to be assessed.

## Results

### Response rate and PREs

**Fig. 1:**
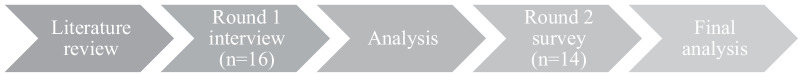
Delphi process

Of the 26 experts approached, 13 agreed to participate. Additional snowball sampling undertaken by PREs resulted in a total of 16 PREs. These PREs came from 11 organisations and included academic researchers in prejudice reduction, racism, and intergroup processes using experimental and qualitative techniques; individuals who had personally delivered, scaled up, and / or evaluated prejudice reduction or anti-discrimination training to large organisations; government policymakers and program officers in multicultural affairs and anti-discrimination; and practitioners working within community groups to deliver community-based prejudice reduction programs for over a decade (Table 2). Nearly all participants were at senior management level, CEOs or equivalent, or were at senior academic levels. The PREs all had direct experience in the decision-making process within their organisation on the scaling of interventions and programs. Many PREs had worked across sectors so have been classified according to their primary sector but care was taken in the synthesis to classify themes according to their relevant sector of experience rather than by the primary sector of the PRE. Although participants were predominantly based in Australia, with only one participant based in the US, many had international experience including in research programs and in delivering programs as part of an international network. In the second round, each organisation provided feedback but only 14 out of 16 participants responded (88%) (Fig. 1).

**Table 2:** PREs by organisation type

Organisation type	Total PREs (n = 16)	Number of organisations
Government	7	4
Community practitioners	4	3
Academic	3	2
Non-government	2	2

### Definition of scalability

In the first-round interviews, PREs were asked for a definition of “scalability”. Their responses mostly focused on concepts of reach and impact but also implicitly indicated that the program or intervention needed to be effective as well. Based on these responses, the following definition was proposed:

Scalability is the capacity of programs and interventions, already shown to be effective, to increase in reach and impact.

This definition was tested in the second-round survey with the PREs, particularly to understand how they defined the key terms “effective”, “reach” and “impact”. Following the feedback from the second round and considering that nearly all PREs felt that impact and effectiveness were synonymous, the final definition of scalability was formed:

Scalability is the capacity of programs and interventions to increase in reach and impact.

At a high level, the PREs’ definition of “impact” could be summed up as “having a concrete and demonstrable effect on people’s lives” or in other words “actual change”. This could be broken down further into two components. The first was *improving key outcomes*—the PREs highlighted a need for a measurable change in target outcomes. The target outcomes included changes in behaviour, attitudes, engagement, and awareness in the broader community. The second component was *degree / effect size*, that is the size of the change.

According to the PREs in this study, reach only mattered if there was impact. The most common type of reach identified by the PREs was the replication of a program with a different target group. This referred to scaling a program to a different state, suburb, school-age group, community, council etc., with some adaptations to the new context to fit new target groups. PREs also viewed the increase in take-up of a program within the same group as reach. Examples of this form of reach included additional people signing up to a program with no change in eligibility criteria or more people accessing the same information on a website. This type of reach was particularly relevant to those PREs who had a national target audience. However, there was a dissenting view that suggested that this type of reach is growth rather than scaling because it is the improvement of targeting a program to a primary audience.

### Key factors for successful scaling

The round 1 interviews with PREs revealed insights that were then developed and tested with the same group in the round 2 survey. The feedback received in the round 2 survey focused predominantly on definitions of impact and effectiveness. Other feedback raised specific points of clarification or scope of the proposed themes. No new themes were proposed through round 2 indicating the process had reached consensus.

Based on this approach, we arrived at 10 high-level assessment factors which we grouped into intervention, delivery, costs, and context categories (Table 3). These factors are the main themes revealed by the PREs. Given the complexity of the “real world”, the factors are likely to be interconnected and there could be minor influences that we have not captured. However, given the aim of this study was to develop a manageable practical tool, the focus has been on capturing the major influences on scalability to inform our framework for assessing scalability rather than to exhaustively document all influences on scalability and their linkages.

**Table 3:** Framework of scalability criteria for prejudice reduction interventions

Category	Theme	Sub-theme	Detail or example
Intervention	Research and evidence use	Application of research and evidence in intervention—evidence-based design	Access to evidence
	Effectiveness	Impact of the intervention on prejudice	Variable approach to effectiveness
	Acceptability	Level of engagement and acceptability with target audience	Tailoring to participant needs
	Format	Suitability of format based on capabilities, costs and target audience	Online vs in-person
Costs	Costs and resourcing	Funding constraints	Source of funding and resources identified and cost-effectiveness
Delivery	Feasibility and provider capacity	Internal physical constraints on scaling	Staff capacity at scale
	Legal	Intellectual property constraints on scaling	Terms of collaboration
	Adaptability	Adaptability of intervention to specific context and practical realities	Tailoring required
Context	Stakeholder buy-in	Political stakeholders	High profile events
		Organisational	Cause champions / leaders and organisational imperatives; provider reputation
		Community stakeholders	Role models
	General context	Societal dynamics	Intergroup conflict, language, sentiment

#### Intervention-related factors

Intervention related factors refer to the design of the prejudice reduction programs and interventions. Each of these are detailed below.

##### Research use and evidence base

While the PREs generally agreed that research and evidence should be core in program development, they also indicated that the scaling of research into practice is met with challenges, in particular accessing academic research due to the lack of “approachable” format of the research. *“Research reports that are 60 pages long are kind of useless… Most people are not going to read that….”* To overcome these challenges, many PREs suggested the need for ongoing engagement and collaboration across sectors. This often occurred through practitioners developing an informal network of academic connections.

##### Effectiveness

Effectiveness was important, with PREs agreeing there was no point in scaling a program or an intervention that did not demonstrate impact. Yet there was no consensus as to how effectiveness should be determined. Some academic PREs indicated a need to focus research on what works in the field rather than laboratory settings. Practitioner PREs indicated impact and effect sizes of interventions were informally evaluated. The concept of effectiveness appears to be quite variable and does not necessarily align with academic norms on evaluation practices.

##### Acceptability to target participants (individual community members or employees)

Multiple PREs noted that it was incredibly important that interventions and programs were framed in a way that was acceptable to its participants. An example raised by several PREs was that often prejudice is linked to conservatism and seen as a form of ignorance. This could make it difficult for individuals to admit to being prejudiced, and lead to them feeling threatened. These feelings of threat can lead program participants to disengage or to opt-out altogether. Additionally, participation in an intervention or program could generate fears of reputational damage among organisations if their employees’ participation might uncover prejudice. This factor focuses on the views of the participants as opposed to stakeholders who are not directly participating in the intervention.

##### Format of the intervention

According to the PREs, format choices require a balance between reach and impact. They tended to gravitate towards digital formats, including online training, use of social media and apps, to increase scalability potential.

"*When we started to then think about how do we deliver cultural training to the 120,000 people… We're talking about a substantial amount of money that we would never have been approved… So you know at the time online training was, I think, a key sort of training program.*"

However, others highlighted that digital formats bring a different set of challenges, in particular the challenge of equitable access to technology. Low-income, language, digital literacy, and disability access barriers restrict the reach of a digital intervention. For practitioners, digital formats were also thought to have lower impact. Many indicated a preference for face-to-face delivery despite it being more resource-intensive.

Face-to-face delivery has obvious limitations for scaling. Many PREs noted the difficulty in ensuring program fidelity and quality across multiple locations and multiple trainers. Some PREs argued that too much tailoring can be problematic if it detracts from the essence of the program.

#### Costs and resourcing

Costs and resourcing were frequently mentioned as key considerations for scalability decisions in the round 1 interviews. Costs often would influence choices on which format to adopt. For example, one PRE indicated that *“the online component… that kind of came out of a desire to have training for everyone so to speak, in a cost-effective way”.* The reference to cost-effectiveness was made by several PREs, who indicated that successful scaling needed to weigh up the effectiveness of the program or intervention against the costs incurred.

#### Delivery

A number of factors are related to the implementation of the intervention or program. These included the adaptability of the program, whether there was capacity and feasibility to deliver at scale, and whether the right agreements were in place to support scaling.

##### Adaptability of program

PREs indicated that the adaptability of programs and interventions to particular contexts was critical for scaling. PREs noted that although they might design a program based on the evidence of what works, the reality was that programs might not fit the specific context and practical realities in which they operated. For example, a program might need to be segmented into smaller blocks so that participants can choose smaller units due to time constraints.

##### Feasibility of delivery at scale and provider capacity

The feasibility to implement an intervention at scale was discussed by the PREs. If a key feature of a program included personalised support for members, this may not be feasible at scale as it would exceed a provider’s capacity. Additionally, multiple PREs noted that the absence of specific skillsets and significant experience in prejudice reduction across contexts could create a ‘natural’ limit to scalability. Staff burnout was also viewed as potentially limiting scalability, particularly for contact-based interventions, where minority group staff members meet program participants*.*

##### Restrictive or unclear intellectual property (IP) agreements

Restrictive or unclear IP agreements have placed limits on the ability of some PREs to scale their interventions and programs. In developing interventions collaboratively between academics and practitioners, there were instances where IP agreements did not have the flexibility to adapt and scale the final output or only with the consent of all parties. Where parties did not have the same vision for the scaling of their intervention, joint ownership was restrictive on the future use of the intervention.

#### Contextual factors

Context was frequently cited as a key consideration in the round 1 interviews. Nearly all PREs noted the importance of stakeholder buy-in. PREs broadly considered three types of stakeholders: political, organisational, and community. General context issues such as social dynamics and cultural, linguistic, and other aspects were also raised by the PREs. This split between factors relating to stakeholders and factors relating to the general context parallels the general theoretical foundations of prejudice. Prejudice theoretically can arise both from individual (e.g., personality and cognition) and collective drivers (e.g., culture, traditions, stereotypes). While these individual and collective factors are interrelated, for the purpose of assessing scalability the PREs considered them separately.

##### Political stakeholders

Many PREs from the community and public sectors commented that politicians ultimately make the funding decisions. Political constraints limit what public organisations can effectively achieve in prejudice reduction strategies. Many PREs noted that the role of politics is quite different when it comes to prejudice reduction as it is an issue that attracts significant political partisanship and debate. Political decision-makers do support large-scale programs that tackle prejudice and discrimination, but as two PREs mentioned, this is often only after a high-profile racist incident.

##### Organisational stakeholders

In the organisational context, the importance of buy-in from managers and executives to the success of scaling prejudice reduction programs was clear:

"*One manager in one of them was ‘Yeah you should really do [the training]. It's really good… And all of her staff were like ‘the training’s awesome. It's really cool.’ … The other [manager] was not like that at all. The other one… was like ‘oh I don't know why we have to do this. Such a waste of time… it's not like we have [minority staff members and clients]. I don't see a problem… That's how the rest of her staff felt as well.*"

Having champions for the cause who are relatively senior decision-makers and creating awareness of benefits helped organisational leaders to build a case for allocating resources to prejudice reduction strategies. The PREs indicated that it is not enough to understand the importance of prejudice reduction in an operating environment where there are competing priorities. For many organisations, prejudice reduction and similar programs are not the core business of the organisation. According to the PREs, these competing priorities can result in limited resources and commitment being allocated to prejudice reduction programs. In the experience of practitioner PREs, a strong reputation could help create trust between the program provider and participants and ensure ongoing demand or interest despite competing priorities.

##### Community stakeholders

Community support was viewed as critical for the scaling of prejudice reduction interventions. Consultation and co-design with communities (through community leaders) helped to achieve buy-in. Through consulting and working with communities to understand needs, programs could be designed to fit the context and purpose. In the broader community, role models were noted as helpful, particularly in delivering messages. Some PREs indicated that sporting figures, high profile individuals and members of minority groups could be effective spokespersons for reaching particular segments of the community and delivering messages.

##### General context

Prejudice reduction programs and interventions operate in specific social contexts. The PREs noted that the context included cultural, linguistic, and religious considerations relevant to a particular community as well as the media narrative, community sentiment and values, and other aspects that create social dynamics of power and norms.

The social dynamics that reflect existing inequalities in society and structural power differences may coincide with the fear of changes in power and privilege and has implications for scalability. According to one PRE,

"*…if you come from a model that locates prejudice much more in intergroup relations and also in contexts that has to do with, ‘I'm feeling threatened as a white person… by the arrival of immigrants’, then it becomes much more complex because that very much depends then on who I am, my power, my relationship to the outgroup, my relationship to the ingroup…*"

The majority of PREs agreed that the context of each community differed and addressing these differences encouraged local buy-in. Understanding and adapting to the local context (or new target population) was universally considered a critical factor for scalability.

### Application of the framework to prejudice reduction interventions

In this section, we apply our framework to a selection of prejudice reduction interventions that have been shown to be effective in field settings. We focus on field experiments in our case studies as they represent the strongest form of evidence for what works in real-world settings, which are the environments where scaling is needed. Experiments have long been used to examine the impact of interventions on prejudice. One of the critical features of an experimental approach is random assignment whereby participants are randomly assigned to receive the “treatment” or to be in a control group because it gives confidence that any difference between the treatment group and the control group is due to the intervention itself, rather than underlying characteristics of the members of the group. This allows causality to be attributed to the intervention. However, interventions that are tested in artificial settings may not necessarily yield the same results in the real world where the context is vastly different [38]. For the purposes of designing a prejudice reduction intervention with the goal of real-world scalability, testing in field environments is of much greater interest. Thus, field experiments are considered to be the most probative type of evidence of what works to reduce prejudice and were the starting principle for our choice of case studies [1].

Within the pool of field experiments that aimed to reduce prejudice, we selected three exemplar studies reflecting key aspects of the prejudice reduction literature, a common form of intervention for each particular theoretical approach, and relatively large effect size within its theoretical approach category according to a recent meta-analysis [19]. Our first case study used a face-to-face contact approach. Contact theory was formalised in the 1950s by Allport and is based on the premise that prejudice between groups can be reduced when members of the two groups meet [39]. It is one of the most commonly tested prejudice reduction approaches in field settings [40]. Notwithstanding this, it has been recognised that there are limitations to the scaling of face-to-face contact interventions as there is a range of difficulties involved in encouraging individuals to engage in intergroup contact [24]. In light of this, the contact literature has electronic forms, which we consider in our second case study. Finally, our third case study used a relatively untested approach of perceived variability—the degree to which an individual views members of the outgroup as heterogeneous [41]—which has been found by some field studies as being highly effective and as outlined in detail below shows good potential for scalability. Through this exercise, we provide a scalability assessment for common forms of popular prejudice reduction approaches as well as a novel approach to draw implications for where research efforts should be concentrated to best tackle prejudice at scale.

#### Example 1: Face-to-face contact intervention [35]

This intervention centred on a class exchange program where primary school students from Israeli-Jewish schools met with students from Israeli-Palestinian schools. Students in the treatment classes were engaged in activities requiring interaction with students from different backgrounds. This consisted of six 4-h monthly meetings. The curriculum included sessions aimed at creating awareness of self and others. Meanwhile, the students in the control condition engaged in art activities without any interaction with students from the other group.

Based on the details provided in the article, there appear to be significant issues with scaling this intervention (Table 4). Applying the scalability factors, the issues that are likely to arise are related to the acceptability of the intervention and physical constraints. Contact is difficult to encourage, in part because it generates anxiety [24]. The face-to-face nature of the contact in this intervention and its requirement for active participation could raise issues with acceptability more so than the more detached approach of an electronic text-based contact intervention (discussed next). Furthermore, the requirement for an adequate number of minority group members could lead to physical constraints for scaling this intervention. The face-to-face format could result in more binding constraints on this factor than compared to an electronic contact format.

**Table 4:** Application of the framework to Berger et al. [35]

Category		Theme	Code	Comments
Intervention	1	Research and evidence use	***	Draws from contact literature
	2	Effectiveness	***	Relatively effective among contact studies, extensive literature on contact—likely to have been replicated but need to confirm
	3	Acceptability	*	Difficulties may arise in encouraging participants to meet; time commitment required unlikely to be acceptable outside of the school environment
	4	Format	**	Time and labour intensive
Costs	5	Costs and resourcing	**	Depends on logistics required for the meeting, requires closer consideration
Delivery	6	Feasibility and provide capacity	*	In-person contact and requirement for minority members creates physical constraints; potential difficulty in maintaining program fidelity
	7	Legal	***	Intervention details accessible via publication
	8	Adaptability	**	Difficult to adapt outside of school context
Context	9	Stakeholder buy-in	-	Insufficient information to assess application in other contexts
	10	General context	-	Insufficient information to assess application in other contexts

Several scalability factors presented more ambiguous challenges. For example, while in-person meetings are likely to be more resource-intensive (e.g., requiring facilitators, meeting rooms, etc.), this may not necessarily be the case if appropriate measures were available (e.g., automated booking systems). Thus, costs and resourcing require closer consideration rather than being a clear constraint on scaling. Similarly, the time-intensive design of the intervention, requiring a total of 24 h of participants’ time could constrain scaling in a non-school setting. Yet there could be situations where this would not be the case. For example, if an employer decided that tackling prejudice is linked to the core business, this could justify the time investment of staff. As there was little information on the factors that relate to why the experiment was successful in that particular context, this could not be assessed in this exercise.

#### Example 2: Electronic contact intervention [36]

This intervention involved pairing undergraduate students to work together on a project. The majority group students (Israelis from European and Latin American backgrounds) were paired with Israeli-Ethiopian students to interact in three online text-chat sessions to co-develop a travel guide. The design featured electronic contact as its mechanism for influencing prejudice.

Based on the details provided in the article, the intervention met some of the scalability criteria (Table 5). The intervention design had demonstrated efficacy in the literature as evidenced by a growing body of evidence on electronic contact and prejudice reduction [42, 43]. However, the time commitment and the degree of interaction required between majority and minority group members could limit the acceptability and adaptability of the intervention to non-student audiences. The intervention required an adequate number of minority group members to pair with which could also constrain scaling.

**Table 5:** Application of the framework to Abu-rayya [36]

Category		Theme	Code	Comments
Intervention	1	Research and evidence use	***	Draws from research literature
	2	Effectiveness	***	Effective results reported, extensive literature on contact—but less in online settings
	3	Acceptability	**	Intervention tested in a number of countries; may be difficult to implement outside of school environment; significant time requirement; electronic contact may be more acceptable than in person
	4	Format	**	May not scale outside of school environment, significant time requirement
Costs	5	Costs and resourcing	**	Leverages common technology e.g., video conferencing or text chat but requires digital access; relatively effective intervention in non-face-to-face contact category; similar to typical school curriculum so costs likely to be considered reasonable
Delivery	6	Feasibility and provide capacity	**	Requires an adequate number of minority group members to pair up in exercise
	7	Legal	***	Intervention details accessible via publication
	8	Adaptability	**	Good potential to adapt contents to other contexts—however, may be difficult to adapt outside of the school environment due to the nature of tasks
Context	9	Stakeholder buy-in	-	Insufficient information to assess application in other contexts
	10	General context	-	Insufficient information to assess application in other contexts

#### Example 3: Perceived variability [37]

This intervention comprised a randomised control trial across eight high schools. In the treatment schools, a poster highlighting the diversity of Arabs was put up in classrooms and outside the school principal’s office for two weeks. In the control schools, no poster was put up. The mechanism used to reduce prejudice was to increase the perceived variability of Arabs (i.e., the perception that Arabs were a heterogeneous group and that individual Arabs differed from each other in various aspects).

Based on the details provided in this article, this intervention met most of the criteria required for scalability (Table 6). The intervention design had demonstrated efficacy in the literature, albeit perceived variability is a relatively new intervention approach and only featured in a handful of studies. The subtle nature of the intervention (i.e., the placement of a poster on the wall) required minimal active engagement on the part of the participant, likely increasing acceptability of the intervention to participants. Further, the poster format was likely to be cost-effective, although scaling to other formats (e.g., billboards, online advertisements) could involve greater costs.

**Table 6:** Application of the framework to Er-rafiy and Brauer [37]

Category		Theme	Code	Comments
Intervention	1	Research and evidence use	***	Draws from contact literature
	2	Effectiveness	**	Large effect size relative to other prejudice reduction field experiments—however, the theoretical approach is based on limited studies
	3	Acceptability	***	Subtle intervention, a poster on wall likely to be acceptable as it does not require active engagement
	4	Format	***	Poster-based intervention is likely to be easy to scale
Costs	5	Costs and resourcing	***	Likely to require minimal cost due to poster format but scaling to advertising boards could require further costs
Delivery	6	Feasibility and provide capacity	***	Poster format is likely to be easy to scale and maintain fidelity
	7	Legal	***	Intervention details accessible via journal publication
	8	Adaptability	**	Relatively untested approach—current studies do not consider effectiveness beyond Arab-Muslims
Context	9	Stakeholder buy-in	-	Insufficient information to assess application in other contexts
	10	General context	-	Insufficient information to assess application in other contexts

One potential limit for scalability could be the adaptability of the intervention. Given that the underlying approach, perceived variability, is still a relatively untested approach there may be unknown constraints in adapting this intervention in field settings to target groups other than Arab Muslims.

## Discussion

Through the input of PREs in the Delphi process, we have arrived at a definition of scalability specific to prejudice reduction and developed a novel framework for scalability specific to prejudice reduction intervention. These findings make a unique and important contribution to the prejudice reduction literature by identifying the factors that are likely to support (or impede) scalability. In particular, the importance of contextual factors was evident in the framework for scaling prejudice reduction interventions.

The resulting framework for scalability of prejudice reduction interventions can be applied to interventions published in the academic literature to identify interventions with the best likelihood for scaling. We illustrated this through the application of the framework developed herein to several published interventions. In doing so, we provide further evidence that commonly used prejudice reduction interventions do not meet all criteria for scalability and may have limited utility in reducing prejudice at scale in broad community settings. We discuss each of these findings in turn below.

### Context requires careful consideration for successful scaling

While for the most part, the factors in the prejudice reduction scalability framework are commonly observed in the scaling frameworks in health, the significance of context for scaling prejudice reduction interventions was uniquely apparent. This was in line with the assessment factors of Zamboni et al. [13]. Context for the successful scaling of prejudice reduction interventions goes beyond the delivery-related factors that Al-Ubaydli et al. [21] proposed. While they alluded, in passing, that representativeness of the situation may include issues such as political opposition, the examples that they focused on were largely related to delivery—whether the delivery, dosage, and program were correct. To successfully scale a prejudice reduction intervention, there is a need for genuine consideration of context, separate from consideration of delivery issues. In our study, the PREs almost universally raised the importance of context or “real-world factors” that a framework grounded in rational economic assumptions would not necessarily include.

Our results from the Delphi process support the view of Supplee and Kane [22] that contextual factors like leadership, relationships with stakeholders and researchers, timing with political and policy windows—the realities of the policy process—need to be addressed. Indeed, as we consulted PREs beyond the government sector, our results go further to suggest that these are relevant across sectors and not just in the policy context.

The challenge then with assessing context is that published research rarely discusses the contextual factors that have contributed to the success of an intervention. In the application of our scalability framework to some exemplar interventions, we could not assess the interventions on contextual factors because of the limited information. Without this information on contextual factors, it is difficult to understand whether an intervention meets the context criteria for successful scaling in other contexts. To optimise scalability, we would encourage greater discussion of the contextual factors and their influence on the impact of the intervention in future research.

### Scalability considerations indicate new directions for prejudice research

In applying our scalability framework to three exemplar interventions, it was evident that some of the more commonly researched approaches to prejudice reduction are less likely to scale. Our framework indicated that a common type of intervention based on face-to-face contact is likely to confront the most difficulty in scaling because of the practical constraints, and also because in-person contact may be less acceptable to participants than more passive approaches. Even a common form of an intervention based on electronic contact may face limitations to scaling due to limitations in access to technology and the required time commitment. Contact is a relatively high-touch intervention type as it requires active participation. Thus, recruitment for the intervention could be difficult as people may be unwilling to participate in an intervention that involves meeting adversaries [24, 25]. The concerns that we raise regarding the scalability of both in-person and electronic contact interventions are based on common forms of those theoretical approaches—it could be that there exist more niche forms that have greater scalability.

This exercise of applying the framework suggests that from a scalability perspective, interventions that are more subtle and able to be published in media, like those based on perceived variability, are much more likely to have the greatest potential for scaling. These interventions avoid the practical hurdles of contact-based interventions as they do not require active participation—participants are not required to interact with outgroup members. Thus, an intervention using a perceived variability approach is more likely to be acceptable to participants who are pre-disposed to prejudice.

However, to date, the greatest effort in understanding how to reduce prejudice has focused on contact-based approaches. Reviews of prejudice reduction interventions demonstrate that the prejudice reduction theory most frequently employed in academia, and often used in practice, is contact theory [1, 39, 44]. As an indication of the vast volume of literature on contact, one of the most comprehensive and structured reviews of the contact-prejudice reduction literature located 515 studies, drawing from 713 independent samples, on the effect of intergroup contact [44]. This focus on contact has not subsided since that review [39, 45].

There has been substantially less research effort on other intervention approaches including those that have a greater possibility of scaling. This is problematic because tackling a problem as pernicious and widespread as prejudice requires interventions that can scale. It is not enough to just know what works or even be the most effective approach. Without the ability to scale, such interventions cannot address the reach of prejudice as a social problem. To broaden the suite of prejudice reduction interventions, research into what works to reduce prejudice needs to expand from contact-based approaches towards intervention approaches that have greater potential to scale.

### Limitations

While there were strengths to the Delphi approach in the development of the scalability framework for prejudice reduction, such as the depth of commentary and expertise that it solicited, this depth did come at a limit to breadth. For this study, we used a small number of PREs and although our sample size was similar to that in studies using a similar method, we do recognise that this is a limitation that is inherent in the Delphi approach. We believe that our results are comprehensive given the substantial expertise represented by the PREs. Furthermore, the Delphi approach is considered to be a valid research method and has been applied extensively to produce rigorous results, especially when participants bring substantial expertise on the matter [12, 27]. We also recognise that even though many of the PREs had international experience and are internationally recognised as experts in their fields, the PREs at the time of participating were largely based in Australia. In light of this, we suggest that the next step for our prejudice reduction framework would be to test this framework in real-world settings with a broad set of users to refine its application across many settings and countries. While it was beyond the scope of our study to explore in-depth the interconnectedness of the factors, further research with this focus could be conducted using our scalability framework to assess a large number of interventions to develop a detailed mapping of the interconnectedness of the sub-factors and themes.

Our choice of exemplar interventions to illustrate the application of the scalability framework sought to demonstrate potential variation in scalability across common forms of different theoretical approaches. We focused on examples with relatively large effect sizes within each theoretical approach according to a recent meta-analysis of prejudice reduction field experiments [19]. Future studies may wish to take a different approach to selecting suitable exemplars based on broader criteria.

We also narrowly defined the scope of the study to only prejudice reduction interventions but noticed that many of the factors are quite general. The more general factors could potentially have broader application beyond prejudice reduction into other social programs. Future work may consider whether this framework applies to other social programs.

## Conclusion

Using a two-round Delphi process, we developed a framework that has considerations for successfully scaling prejudice reduction interventions, based on the insights and experience of PREs across the discipline. The scalability framework is the first to be developed and applied to prejudice reduction interventions. By identifying the best practice principles for scalability, the framework has applications beyond advancing academic knowledge. These principles provide necessary guidelines to assist policymakers or practitioners in their selection or design of a scalable intervention for reducing prejudice. The framework provides guidance on considerations of practical issues and complements the issues relating to the applicability of the evidence base proposed in theoretical models. In doing so, the framework guides the user to have a better understanding of whether factors that lead to scalability are substantially met. This better understanding of scalability also revealed that more research effort needs to be been placed on prejudice reduction approaches with a greater possibility of scaling to better tackle the pervasive problem of prejudice in society.

## Data Availability

The datasets generated and/or analysed during the current study are not readily available to protect participants privacy and confidentiality requirements as part of ethics approval. Requests to access the data should be directed to the corresponding author.

## Abbreviation

PRE: Prejudice reduction expert
